# Roles of WRKY Transcription Factors in Response to Sri Lankan Cassava Mosaic Virus Infection in Susceptible and Tolerant Cassava Cultivars

**DOI:** 10.3390/plants14081159

**Published:** 2025-04-08

**Authors:** Somruthai Chaowongdee, Nattachai Vannatim, Srihunsa Malichan, Nattakorn Kuncharoen, Pumipat Tongyoo, Wanwisa Siriwan

**Affiliations:** 1 Department of Plant Pathology, Faculty of Agriculture, Kasetsart University, Bangkok 10900, Thailand; somruthai.ch@ku.th (S.C.); nattachai.va@ku.th (N.V.); srihunsa.m@ku.th (S.M.); nattakorn.ku@ku.th (N.K.); 2 Center of Excellence on Agricultural Biotechnology (AG-BIO/MHESI), Bangkok 10900, Thailand; pumipat.tong@ku.th; 3 Center for Agricultural Biotechnology, Kasetsart University, Kamphaengsaen Campus, Nakhon Pathom 73140, Thailand

**Keywords:** WRKYs, plant defense mechanisms, *Sri Lankan cassava mosaic virus*, phenotypic variations, plant defense mechanisms

## Abstract

Cassava mosaic disease (CMD) is caused by viruses such as *Sri Lankan cassava mosaic virus* (SLCMV). It poses a significant threat to the cassava (*Manihot esculenta*) yield in Southeast Asia. Here, we investigated the expression of WRKY transcription factors (TFs) in SLCMV-infected cassava cultivars KU 50 (tolerant) and R 11 (susceptible) at 21, 32, and 67 days post-inoculation (dpi), representing the early, middle/recovery, and late infection stages, respectively. The 34 identified WRKYs were classified into the following six groups based on the functions of their homologs in the model plant *Arabidopsis thaliana* (AtWRKYs): plant defense; plant development; hormone signaling (abscisic, salicylic, and jasmonic acid); reactive oxygen species production; basal immune mechanisms; and other related hormones, metabolites, and abiotic stress responses. Regarding the protein interactions of the identified WRKYs, based on the interactions of their homologs (AtWRKYs), WRKYs increased reactive oxygen species production, leading to salicylic acid accumulation and systemic acquired resistance (SAR) against SLCMV. Additionally, some WRKYs were involved in defense-related mitogen-activated protein kinase signaling and abiotic stress responses. Furthermore, crosstalk among WRKYs reflected the robustly restricted viral multiplication in the tolerant cultivar, contributing to CMD recovery. This study highlights the crucial roles of WRKYs in transcriptional reprogramming, innate immunity, and responses to geminivirus infections in cassava, providing valuable insights to enhance disease resistance in cassava and, potentially, other crops.

## 1. Introduction

Cassava mosaic disease (CMD) is a major concern in cassava (*Manihot esculenta*) plantations in Southeast Asia. It is caused by *Sri Lankan cassava mosaic virus* (SLCMV) (genus *Begomovirus*, family *Geminiviridae*), which has a single-stranded DNA genome and a twinned icosahedral particle morphology [1,2,3,4,5]. It was first reported in Sri Lanka and India and then spread to other countries via SLCMV-infected stem cuttings [1,5,6,7]. The first official report of SLCMV in Southeast Asia was in 2016 [6]. Thailand is one of the largest cassava exporters in the world, and due to the emergence of CMD in cassava plantations, Thailand’s cassava farmers and processing companies have faced severe challenges in terms of low cassava production efficiency and large economic losses. CMD has had a devastating impact on cassava production, causing yield losses and physical disorder, including growth disruption, stunning, reduced flour yields, smaller tuber size, and in severe cases, complete crop failure. Unfortunately, knowledge on SLCMV and its host plant is currently insufficient; more research is needed on breeding resistant and tolerant cultivars, which is the most efficient strategy to control SLCMV.

Cassava phenotypes include resistant, tolerant, and susceptible phenotypes, reflecting their responses to viral infection [8,9,10,11]. The Kasetsart 50 (KU 50) cassava cultivar is a well-known cultivar in Southeast Asia due to its tolerance to SLCMV infection, meaning that it can tolerate the virus without developing severe symptoms. On the other hand, the Rayong 11 (R 11) cassava cultivar has a susceptible phenotype, exhibiting clear leaf symptoms [12]. Knowledge of the genes that regulate casava’s defense mechanisms is key to understanding the transcriptional responses to viral infections and other stresses.

Transcription factors (TFs) are unique regulatory proteins in eukaryotic cells that regulate gene expression at the transcriptional level by binding to specific DNA regions in target gene promoters and activating or repressing transcription [13,14,15]. Plant TFs belong to about 58 reported families [16,17]. Some of these TFs participate in plant innate defense, including signaling pathways related to pattern-triggered immunity (PTI) and effector-triggered immunity (ETI) [16,17,18].

The WRKY family is a large family of TFs [19]. They are implicated in the transcriptional reprogramming that occurs as part of immune responses in plants, acting as positive or negative regulators of disease resistance [20]. WRKYs can be categorized into three major groups (1, 2, and 3) [21,22,23], this categorization is supported by the classification of WRKYs identified in genome-wide analyses of plants such as cassava (*Manihot esculenta*), maize (*Zea mays* L.), tomato (*Solanum lycopersicum*), rice (*Oryza sativa*), and *Nicotiana benthamiana* [21,24,25,26,27].

In *Arabidopsis*, it has been found that WRKYs respond to bacterial infections and the signaling hormone salicylic acid (SA) [28], and WRKYs also regulate both SA- and jasmonic acid (JA)-dependent defense signaling and mediate the interplay between these antagonistic pathways [29]. Northern blotting and microarray hybridization results indicate that WRKYs can enhance or reduce SA accumulation, pathogen resistance, and constitutive expression of pathogenesis-related (*PR*) genes, which are factors that are indicative of constitutive systemic acquired resistance (SAR) [30].

In this study, we identified *WRKY* family members in a transcriptome analysis of SLCMV-infected cassava cultivars and predicted their functions by identified homologs in the well-annotated model plant *A. thaliana*. This study provides an overview of WRKY expression profiles following SLCMV infection, offering insights into the role of these TFs in plant defense mechanisms. The findings contribute to a deeper understanding of cassava’s response to SLCMV and pave the way for future research.

We explored the defense mechanisms of KU 50 (tolerant) and R 11 (susceptible) cassava cultivars at three time points—21, 32, and 67 days post-inoculation (dpi)—representing the early, middle/recovery, and late stages of infection, respectively. Our results enhance understanding of WRKY TFs in regulating gene expression crosstalk and protein interactions during plant defense responses. Moreover, these findings could support further studies on SLCMV–cassava interactions and contribute to cassava breeding programs aimed at improving disease resistance.

## 2. Results

### 2.1. Identification of WRKYs in SLCMV-Infected KU 50 and R 11 at 21, 32, and 67 dpi

**Figure 1:**
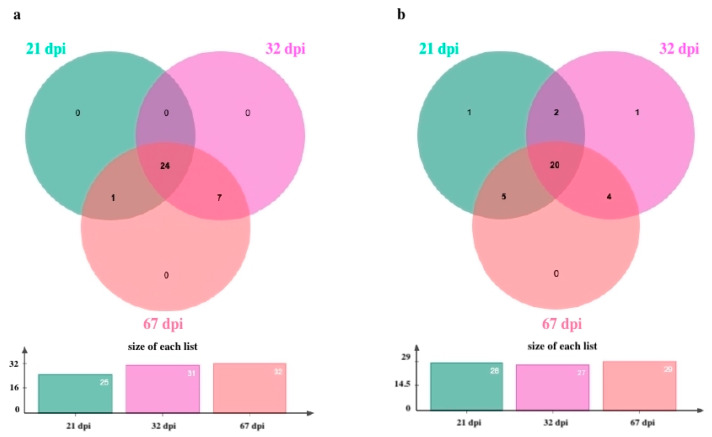
Venn diagrams of all identified WRKYs in this study, which involved 34 WRKYs identified at 21, 32, and 67 dpi in SLCMV-infected (**a**) KU 50 and (**b**) R 11.

As shown in Figure 1, 34 expressed members of the *WRKY* TF family (32 in KU 50 and 33 in R 11) were identified. The identified *WRKY*s, their *AtWRKY* homolog ontology, and their functional categorization are listed in Table 1. There was only one uniquely expressed *WRKY* (*WRKY22*; XM_021762774.1) in KU 50 and two uniquely expressed *WRKYs* (*WRKY43*, XM_021756782.2; *WRKY75*, XM_021743059.2) in R 11.

In KU 50, 24 *WRKYs* were expressed at all three time points, none were uniquely expressed at any specific time point, 7 (*WRKY2*, XM_021757679.2; *WRKY17*, XM_021741844.2; *WRKY31*, XM_021742177.2; *WRKY33*, XM_021767775.2; *WRKY40*, XM_021772630.2; *WRKY44*, XM_021765491.2; *WRKY53*, XM_021748704.2) were expressed at both 32 and 67 dpi, and 1 (*WRKY1*, XM_021774985.2) was expressed at both 21 and 32 dpi (Figure 1a).

In R 11, 20 *WRKYs* were expressed at all three time points, 1 (*WRKY1*, XM_021774985.2) was uniquely expressed at 21 dpi, 1 (*WRKY26*, XM_021749661.2) was uniquely expressed at 32 dpi (Figure 1b), 2 (*WRKY43*, XM_021756782.2; *WRKY44*, XM_021765491.2) were expressed at both 21 and 32 dpi, 5 (*WRKY2*, XM_021757679.2; *WRKY24*, XM_021751071.2; *WRKY27*, XM_021759593.2; *WRKY28*, XM_021742749.2; *WRKY33*, XM_0f21767775.2) were expressed at both 21 and 67 dpi, and 4 (*WRKY17*, XM_021741844.2; *WRKY40*, XM_021772630.2; *WRKY53*, XM_021748704.2; *WRKY70*, XM_021761805.2) were expressed at both 32 and 67 dpi (Figure 1b).

**Figure 2:**
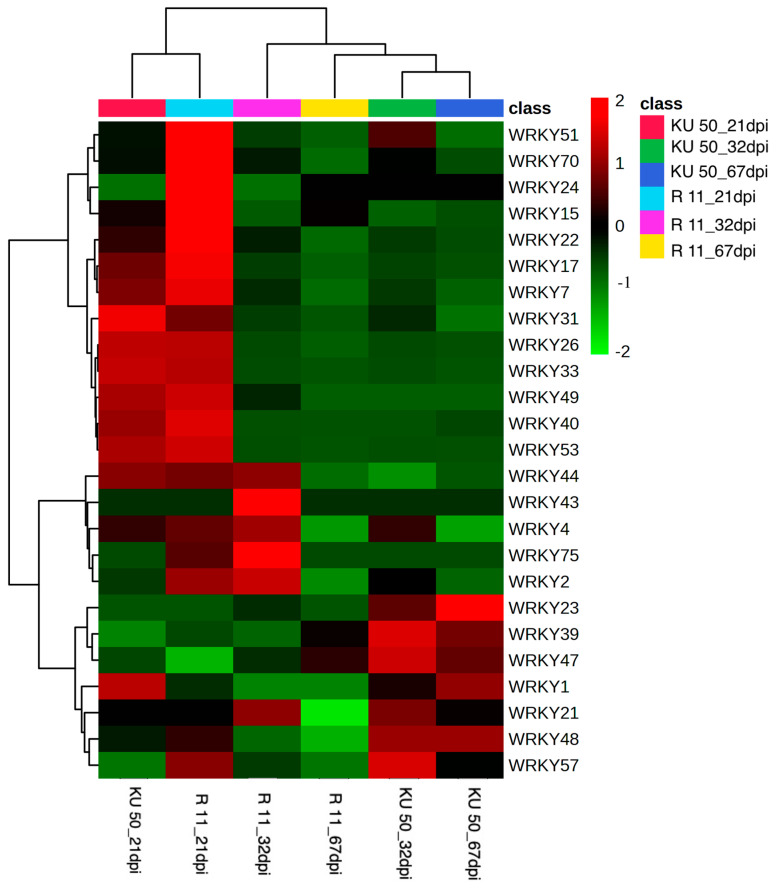
Heatmap of identified WRKYs by cultivar and SLCMV infection stage based on Fisher’s least significant difference tests (*p* < 0.05).

The *WRKY* expression levels are displayed in a heatmap in Figure 2. The heatmap indicates that there were two distinct clusters: (1) KU 50 and R 11 at 21 dpi and (2) R 11 and KU 50 at 32 and 67 dpi. Additionally, the WRKYs were grouped into two main clusters. The first cluster comprised *WRKY51*, *70*, *24*, *15*, *22*, *17*, *7*, *31*, *26*, *33*, *49*, *40*, and *53*, while the second cluster comprised *WRKY44*, *43*, *4*, *75*, *2*, *23*, *39*, *47*, *1*, *21*, *48*, and *57.*

### 2.2. Functions of Identified WRKYs and Phylogenetic Analysis

A phylogenetic tree of the identified WRKYs and *A. thaliana* homologs (AtWRKYs) was constructed based on the phylogenetic tree reported by Wang et al. (2023) [59]. AtWRKY amino acid sequences are categorized based on the zinc finger motif, i.e., groups 1, 2, and 3, with five subgroups for group 2 (2a–e) [21,22,31]. Group 1 and 2 WRKYs have a C_2_H_2_ zinc finger motif, whereas group 3 WRKYs have a zinc finger-like motif ending with C_2_HC [21,22,23]. Thus, our identified WRKYs were also categorized using this classification.

**Figure 3:**
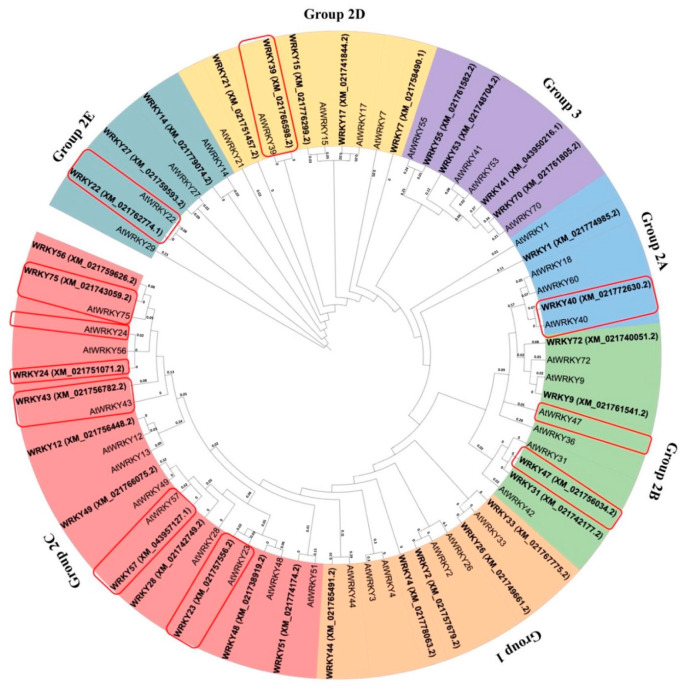
Phylogenetic tree of *Arabidopsis thaliana* WRKYs (AtWRKYs) and the identified WRKYs (with NCBI accession numbers) based on amino acid sequence alignment. The tree was constructed using the NGPhylogeny platform and adjusted using the Interactive Tree of Life (ITOL) tool (bootstrap values were superimposed on branches with <50% support). Classification into three groups (groups 1, 2 and 3), with five subgroups for group 2 (2a–e), was primarily based on the zinc finger motif [21]. Red boxes represent nine key WRKYs that were selected based on the DEG analysis results.

The largest subgroup was group 2c (WRKY12, -23, -28, -29, -43, -48, -51, -57, and -75), and the homologs in *A. thaliana* were AtWRKY12, -13, -23, -24, -28, -43, -48, -49, -51, -56, -57, and -75. Group 1 (WRKY2, -4, -44, -26, and -33) homologs were AtWRKY2, -3, -4, -26, -33, and -44. Group 2d (WRKY7, -15, -17, -21, and -39) homologs were AtWRKY7, -15, -21, and -39. Group 3 (WRKY41, -53, -55, -56, and -70) homologs were AtWRKY41, 53, 55, and -70. Group 2b (WRKY31, 47, 72, and 9) homologs were AtWRKY9, -31, -36, -42, -47, and -72. Group 2a (WRKY1, -18, and -40) homologs were AtWRKY1, -18, -40, and -60. Group 2e (WRKY14, -22, and -27) homologs were AtWRKY12, -22, -27, and -29 (Figure 3).

We also predicted the functions and interacting partners of WRKYs based on those of the AtWRKYs based on the alignment of WRKY amino acid sequences obtained from the NCBI database and The *Arabidopsis* Information Resource (TAIR). The identified WRKYs and AtWRKYs were grouped into certain taxonomic clusters in the phylogenetic tree (Table 1). The WRKYs were categorized into six groups based on their predicted functions (Table 1): (1) plant defense; (2) plant development; (3) hormone signaling (abscisic acid, SA, and JA); (4) reactive oxygen species (ROS) production; (5) basal immune mechanisms; and (6) other related hormones, metabolites, and abiotic stress responses.

Interestingly, 15 WRKYs (WRKY1, -4, -9, -14, -27, -28, -31, -44, -47, -48, -51, -53, -55, -57, and 72) were predicted to be involved in SA and JA signaling. Furthermore, three of these WRKYs (WRKY31, -47, and -55) were also predicted to be involved in ROS production (involving H_2_O_2_ and peroxidase enzymes) and induction of plant programmed cell death. Moreover, 14 WRKYs (WRKY1, -7, -9, -12, -21, -23, -24, -26, -27, -33, -48, -49, -70, and -72) were predicted to be involved in basal immune mechanisms. Other WRKYs may play various roles in the defense against SLCMV infection, including six WRKYs (WRKY2, -17, -26, -33, -39, and -56) that respond to abiotic stresses such as heat stress and soil acidity (Table 1).

According to the DEG analysis, *WRKY43* and *-75*, which were uniquely expressed in R 11 (susceptible), were both upregulated at 32 (21 to 32 dpi) but downregulated at 67 dpi (32 to 67 dpi). Both WRKY DEGs were predicted to be involved in the ABA signaling pathway, indicating a role for some WRKY DEGs in the response to SLCMV in R 11.

### 2.3. WRKY DEGs at 32 and 67 dpi in KU 50 and R 11

**Figure 4:**
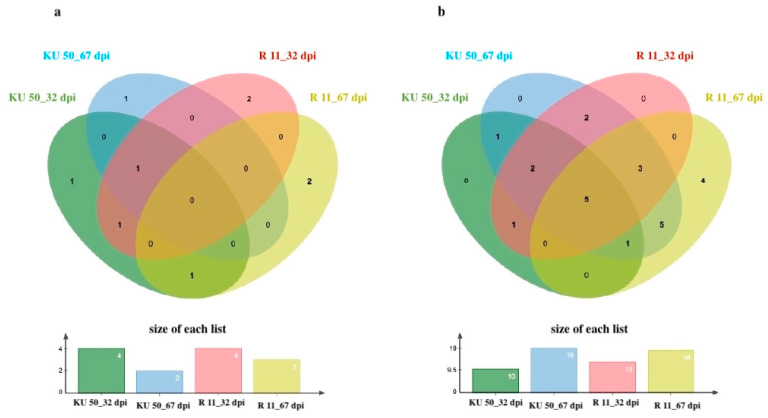
Venn diagrams of (**a**) upregulated and (**b**) downregulated differentially expressed genes (DEGs; *p* < 0.01 and log_2_(fold change) ≤ 1.0) at 32 and 67 dpi in SLCMV-infected KU 50 and R 11.

There were a total of 34 *WRKY*s analyzed (*p* < 0.01 and log_2_(fold change) ≤ 1.0) at 32 dpi (21 to 32 dpi, i.e., early to middle infection stages) and 67 dpi (32 to 67 dpi, i.e., middle to late infection stages) in KU 50 and R 11 (Table 2). The *WRKYs* that were identified in our raw data analysis but not expressed in certain cultivars/stages were labeled with “ND” (not determined) in the DEG count table (Table 2). The *WRKY* DEGs primarily exhibited downregulation as presented as 24 genes were downregulated whereas 9 genes were upregulated) (Figure 4), and the expression of many *WRKYs* varied remarkably by cultivar and time point.

Several WRKYs exhibited unique expression patterns. Notably, *WRKY57* (XM_043957127.1, regulates JA signaling) was uniquely upregulated at 32 dpi in KU 50 (and downregulated at 67 dpi in KU 50 and at both time points in R 11). *WRKY47* (XM_021756034.2, involved in SA signaling and ROS production) was upregulated at 32 dpi and downregulated at 67 dpi in both cultivars.

Additionally, *WRKY39* (XM_021766598.2, associated with heat stress response) was downregulated in KU 50 at 67 dpi and in R 11 at 32 dpi. Furthermore, *WRKY23* (XM_021757556.2, responsive to auxin hormones) was upregulated at both time points in KU 50 but only at 32 dpi in R 11 (downregulated at 67 dpi). Moreover, *WRKY40* (XM_021772630.2, involved in ABA signaling) was uniquely upregulated at 67 dpi in KU 50, downregulated at 32 dpi in KU 50, and at both time points in R 11.

*WRKY43* (XM_021756782.2, involved in salt stress tolerance) and *WRKY75* (XM_021743059.2; involved in phosphate accumulation related to plant growth and development) were upregulated at 32 dpi and downregulated at 67 dpi in R 11 but absent in KU 50. In contrast, *WRKY15* (XM_021776299.2, enhances plant metabolites) and *WRKY24* (XM_021751071.2, involved in salt stress response) were uniquely upregulated at 67 dpi in R 11 and downregulated at 32 dpi in R 11 and at both time points in KU 50. Notably, these genes exhibited different expression patterns in R 11 compared to KU 50 at various time points, indicating their importance in stress response mechanisms.

Four WRKYs (*WRKY43*, XM_021756782.2; *WRKY75*, XM_021743059.2; *WRKY23*, XM_021757556.2; *WRKY40*, XM_021772630.2) were downregulated at 67 dpi in R 11. They are involved in salt stress tolerance, phosphate accumulation related to plant growth and development, and auxin response and ABA signaling, respectively (all categorized in the “other related hormones, metabolites, and abiotic stress responses” group). Interestingly, *WRKY22* (XM_021762774.1, associated with mitogen-activated protein kinase (MPK) signaling and increases the H_2_O_2_ level) was downregulated at both time points in KU 50.

### 2.4. RT-qPCR Validation

The expression of nine selected WRKYs (*WRKY22*, *-23*, *-24*, *-39*, *-40*, *-43*, *-47*, *-57*, and -*75*) was validated using RT-qPCR to quantify the RNA abundance in each sample and then comparing the results to the DEGs derived from the RNA-seq analysis. *WRKY22* was selected as it was uniquely expressed in KU 50 based on RNA-seq; *WRKY43* and -*75* were uniquely expressed in R 11 based on RNA-seq; and *WRKY57* was upregulated in KU 50 at 32 dpi based on RNA-seq.

The comparison of the *WRKY* expression based on RNA-seq and RT-qPCR (log2^−∆CQ^) at 32 dpi (21 to 32 dpi) and 67 dpi (32 to 67 dpi) in KU 50 and R 11 is shown in Table 3. Notably, at 32 dpi in R 11, *WRKY24* was downregulated according to the RNA-seq data but upregulated according to the RT-qPCR data. Conversely, at 67 dpi in R 11, *WRKY24* was upregulated according to the RNA-seq data but downregulated according to the RT-qPCR data. For, *WRKY57* at 32 dpi was downregulated in R 11 and upregulated in KU 50 according to the RNA-seq data, whereas the RT-qPCR data revealed that its expression at 32 dpi was highest in R 11 (expression level: 1.20) and lowest in KU 50 (expression level: -0.32) (Table 3). *WRKY43*, which was uniquely expressed in R 11 according to the RNA-seq data, was upregulated at 32 dpi and downregulated at 67 dpi according to the RNA-seq data but downregulated at both time points according to the RT-qPCR data. In summary, the RT-qPCR results validated the accuracy of the *WRKYs* assessed using RNA-seq.

## 3. Discussion

### 3.1. Exploring Functions of WRKYs

WRKYs expressed in KU 50 and R 11 at 21, 32, and 67 dpi were identified by BLASTn version 2.9.0 searches of RNA-seq data against the *M. esculenta*_v8 NCBI database. Their functions were predicted based on the functions of well-annotated AtWRKYs homologs. Phylogenetic analysis of the identified WRKYs and the AtWRKYs, based on amino acid sequences, showed that the identified WRKYs were grouped into certain taxonomic clusters. Several novel WRKY amino acid sequences were identified and integrated into the phylogenetic tree alongside the previously identified AtWRKY amino acid sequences. The clustering of the WRKYs in the phylogenetic tree was reflective of their presence in susceptible and/or tolerant cultivars. The predicted functions highlight the significant role played by WRKYs in various plant species, especially cassava, including their influence on plant defense responses.

*WRKY1* expression was absent at 67 dpi (late stage) in both cultivars. Downregulation of *AtWRKY1* (a *WRKY1* homolog that suppresses SA signaling) leads to SA accumulation, whereas *AtWRKY1* overexpression reduces the SA-mediated defense response [37]. AtWRKY1 binds to the *PR1* promoter in yeast cells, inhibiting *PR1* transcription [60]; this downregulation of *PR1* decreases resistance against several phytopathogens [60]. Therefore, *AtWRKY1* regulates plant defense responses by controlling plant immune mechanisms such as *PR1* transcription control and SA signaling. This suggests a generally negative regulatory role for *WRKY1* at 32 dpi in both cassava cultivars. *AtWRKY1* is a homolog of *MeWRKY65* and *-29*, and they are involved in plant defense and response to viral infection [21,23].

*WRKY70* was absent at 21 dpi (early stage) in R 11 but was observed at all three time points in KU 50, indicating positive regulation in the tolerant cultivar at the early stage of infection. Li et al. (2004) [61] hypothesized that *WRKY70* is an activator of SA-responsive genes and a repressor of JA-responsive genes, balancing the signals regarding these two pathways. *WRKY70* was downregulated in an SACMV-infected susceptible cultivar (T200) at the middle and late stages (32 and 67 dpi) [62]. During SAR, *AtWRKY70* modulated *NONEXPRESSOR OF PATHOGENESIS-RELATED 1 (NPR1)* expression, and it was associated with *PR1* upregulation [63]. The SAR mechanism is regulated by SA, which influences *PR* expression and promotes plant defense responses, including resistance against virulent pathogens [64,65]. *NPR1* indirectly mediated the SA signaling [66,67,68]. *AtWRKY70* is a homolog of *MeWRKY18*, *-59*, and *-83* [21,23], which positively regulated programmed cell death in SACMV-infected cassava cultivar TME3 (tolerant) at 32 dpi and T200 (susceptible) at 67 dpi [23]. These three homologs are involved in SACMV-induced CMD symptoms by controlling the SA- and JA-dependent pathways [23,62], and MeWRKY83 also affects the ABA-dependent pathway, reducing ABA accumulation under drought stress [69]. Therefore, the absence of *WRKY70* at 21 dpi in R 11 reflects a key difference between R 11 (susceptible) and KU 50 (tolerant) cultivars, with R 11 lacking a basal immune response during early-stage SLCMV infection, while KU 50 activates plant immunity early to restrict SLCMV.

*WRKY43* was uniquely found in R 11 at 21 and 32 dpi and was absent in KU 50 at all three time points. This gene is a homolog of *AtWRKY43* and related to *AtWRKY24* and *-56*. In *A. thaliana*, *WRKY43* positively regulated ABA-dependent gene expression [70]. *WRKY56* expression and functions reflect the expression and function of *AtWRKY56* in *A. thaliana* clones, which was strongly increased as a result of NaCl treatment [58]. *AtWRKY56* is a homolog of *MeWRKY25*, *-31* and *-56* [21]. The functional differences in homologous genes between R 11 and KU 50 suggest another key difference between R 11 and KU 50, beyond their responses to SLCMV infection.

### 3.2. WRKY DEGs Determinations

The *WRKY* DEGs were up- or downregulated in response to SLCMV infection at 32 dpi (21 to 32 dpi) and 67 dpi (32 to 67 dpi). Several WRKYs were expressed at both 32 and 67 dpi in KU 50 (tolerant), while transient expression occurred at either 32 or 67 dpi in R 11 (susceptible). For instance, *WRKY22* (uniquely expressed in KU 50) was detected as downregulated at both time points in KU 50, while *WRKY33* and *WRKY26* were detected as downregulated at 67 dpi in both cultivars. The latter two positively regulated the cooperation of heat shock protein-related signaling pathways that mediate heat stress responses, and they have overlapping thermotolerance functions in *A. thaliana* [52]. In *Arabidopsi*s, *WRKY33* overexpression decreased resistance against *Botrytis cinerea* and *Alternaria brassicicola* infection but increased resistance against *Pseudomonas syringae* infection [51]. *WRKY33* overexpression in necrotrophic fungal infection led to ROS accumulation and the hypersensitive response in susceptible cells, whereas *WRKY33* overexpression in biotrophic bacterial infection enhanced the salicylate-regulated *PR1* expression and then led to resistance [64]. *WRKY33* was absent in R 11 at the middle/recovery stage (32 dpi); the symptoms in R 11 (32 dpi) were detected through perceiving virion particles or diagnosed as viral infection, which accords with the external observation indicating severe disease severity in a similar period. We propose that this may be related to susceptibility to SLCMV infection based on the antagonistic interactions between *WRKY33*-induced defense responses and *WRKY33*-induced susceptibility. Moreover, *AtWRKY33* activates pathogen-associated molecular pattern (PAMP) immunity by regulating the upstream *PAD4* promotor, enhancing SA-independent signaling and SA accumulation, consistent with a plant defense response [70,71,72]. In a study on SLCMV, SA accumulation was detected at 3 dpi in resistant (C33) and 2 dpi in tolerant (KU 50) cassava cultivars but not in the susceptible (R 11) cultivar at any time point (which matched the symptoms of these cultivars) [73]. Additionally, the SA accumulation was decreased immediately after 2 dpi in the tolerant (KU 50) cultivar, which was related to *PR* downregulation, based on RT-qPCR [73]. This supports the role of SA in resistant/tolerant/susceptible phenotypes, involving coordination with *PR*, which affects symptom severity.

*WRKY24* (homolog of *AtWRKY24*, *-43*, *-51*, *-56*, and *-75*) was downregulated at both time points in KU 50 and upregulated at 67 dpi but downregulated at 32 dpi in R 11. In *Oryza sativa* ssp. *Indica*, *OsWRKY24* upregulated early defense response marker genes, such as *NON-RACE-SPECIFIC DISEASE RESISTANCE10* (*NDR10*, homolog of *NDR1/HIN1-LIKE* [*NHL10*]) [74]. *OsWRKY24* accumulation positively modified plant basal immunity (PTI) and increased resistance in rice against the rice blast fungus *Pyricularia oryzae* [50,74]. Thus, *WRKY24* positively regulates immunity in response to SLCMV in R 11 (susceptible) at 67 dpi. This indicates the role and effects of *WRKY24* against viral infections in the susceptible cultivar, but further investigations are required.

Several WRKYs (including *WRKY4*, *-7*, *-9*, *-31*, *-44*, *-47*, and *-55*) were involved in ROS production. However, *WRKY47* was upregulated at 32 dpi in both cultivars, while these other *WRKYs* were downregulated at both time points in both cultivars. *WRKY47* is a positive regulator in the middle/recovery stage (32 dpi) of SLCMV-infected KU 50 and R 11. This indicates a positive response of *WRKY47* expression following the CMD symptomatic stage, along with ROS accumulation at 32 dpi. Regulating ROS accumulation influences various plant defense mechanisms, such as the hypersensitive response and programed cell death to defend against pathogens, including viruses [75]. ROS accumulation directly induces SAR [76], involving sustained *NPR1* promoter binding and pathogen resistance. While ROS accumulation and oxidative stress influence nuclear gene expression and SA and JA signaling, abiotic stress/injury can also upregulate retrograde signaling pathways in chloroplasts, which can stimulate SA, JA, and ROS production [77]. This indicates that WRKYs regulate SA and JA synthesis and ROS production, which may contribute to various defense responses in cassava.

Several WRKYs (*WRKY1*, *-14*, *-24*, *-27*, *-28*, *-31*, *-44*, *-47*, *-48*, *-51*, *-53*, *-55*, *-57*, and *-72*) were predicted to induce the SA and JA pathways. These *WRKYs* were consistently downregulated at both time points in both cultivars, except for *WRKY24*, which was downregulated at both time points in KU 50 but upregulated at 67 dpi in R 11 (Table 2). The crosstalk between JA and SA signaling modulates plant disease resistance against necrotrophic or hemi-biotrophic diseases, with SA inducing initial defense-related gene expression and JA inducing late defense-related gene expression [78,79]. Thus, *WRKY24* may alter the crosstalk between JA- and SA-dependent responses, thereby increasing susceptibility in R 11.

*WRKY22* was uniquely expressed in KU 50, exhibiting downregulation at both time points in this tolerant cultivar. *CsWRKY22* plays a role in susceptibility to *Xanthomonas citri* subsp. *citri* (Xcc) in *Citrus sinensis* (L.) Osbeck [80,81], being upregulated by Xcc in susceptible plants. In contrast, in *A. thaliana*, AtWRKY22 participates in the light response and enhances H_2_O_2_ production [82], indicating its involvement in signaling pathways responding to abiotic stress. Relatedly, *OsWRKY22* overexpression increases *Magnaporthe oryzae* resistance in rice, while *OsWRKY22* silencing increases H_2_O_2_ production and callose accumulation, and it contributes to nonhost resistance against barley powdery mildew in rice [83]. Hence, *WRKY22* may be related to both abiotic and biotic stress responses.

*WRKY15*, which was upregulated at 67 dpi in R 11, induces metabolite production during salt and osmotic stress in *A. thaliana*, which activates mitochondrial retrograde signaling and promotes cellular redox homeostasis [84]. Thus, *WRKY15* may be interpreted as playing a key role in these cellular processes in SLCMV-infected R 11 (susceptible). Further experiments are needed to provide a detailed understanding of these interactions.

*WRKY40* was upregulated at 67 dpi in KU 50 but downregulated at 67 dpi in R 11, indicating that it may act as a positive defense regulator when upregulated in KU 50 at the late stage of infection. *AtWRKY40* regulates ABA signaling in *A. thaliana*, upregulating ABA signaling components. Upregulated *WRKY40* increased SUCROSE NONFERMENTING 1-RELATED PROTEIN KINASE 2 (SNRK2) and phosphorylated SNRK2 then activated RESPIRATORY BURST OXIDASE HOMOLOG PROTEIN F (RBOHF, which is an NADPH oxidase) proteins, which leads to ROS production [34]. In *Arabidopsis* with powdery mildew infection, *AtWRKY18* and *-40* strongly downregulated *JASMONATE ZIM-DOMAIN (JAZ)*, while lack of *AtWRKY18* and *-40* upregulated *JAZ* and downregulated the JA signalling genes [85]. Additionally, *AtWRKY40* is a homolog of *MeWRKY7*, *-9*, *-10*, and *-11* [21], and *MeWRKY11* exhibits positive regulation in a tolerant cultivar, as reflected by its upregulation by SACMV at 32 dpi [23]. This suggests an altered function of the *MeWRKY11* homolog *AtWRKY40*. Our analysis indicated that *AtWRKY40* is involved in the crosstalk between JA and ABA signaling pathways during stress defense and it contributes to the ROS pathway, especially in SLCMV infection. *WRKY40* may increase tolerance in SLCMV-infected KU 50mas it was upregulated at the late infection stage (67 dpi). *WRKY40* may serve as a marker indicating tolerance in SLCMV-infected KU 50.

### 3.3. Interacting Partners of WRKY Proteins

**Figure 5:**
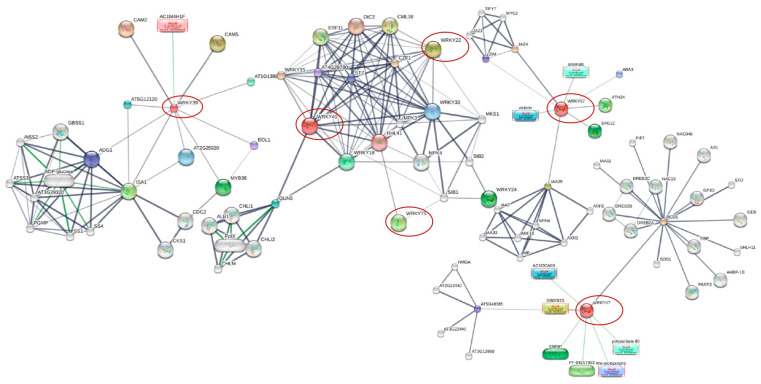
Protein–protein interaction network of WRKYs based on STITCH v5 analysis. Red circles highlight the WRKYs identified in this study.

Our STITCH v5 analysis revealed the interacting partners of the WRKY proteins (Figure 5). Notably, WRKY22, -40, and -75 proteins interact, while others function in separate pathways.

WRKY22, -33, and -40 interact with MPKs such as MPK3 and MPK4. MPK3 and WRKY33 are involved in SAR induction, coordinating pipecolic acid production [86]. MPK3 is also active during PTI and ETI, interacting with nucleotide-binding leucine-rich repeat receptors (NLRs) and enhanced disease susceptibility 1 (EDS1), ultimately contributing to SA signaling [87]. Furthermore, MPK4 interacts with basal immunity components in PTI and ETI signaling pathways in response to biotic stresses [88,89]. These findings underscore the key roles of WRKYs in plant immunity, influencing tolerance/susceptibility.

*WRKY57* (which was notably upregulated at 32 dpi in KU 50 but downregulated at both time points in R 11) interacts with ABA3, the ABA biosynthesis enzyme that is upregulated in response to abiotic stresses such as drought stress in *A. thaliana* [90]. Additionally, WRKY57 interacts with senescence-associated gene 12 (SAG12), a cysteine protease involved in auxin and cytokinin synthesis. Furthermore, WRKY57 interacts with jasmonate-domain zim 4 (JAZ4), a key player in plant defense via its involvement in the JA signaling pathway and regulation of JA-mediated biosynthesis of secondary metabolites [91,92,93]. Notably, 32 dpi (middle/recovery stage) represents a pivotal point during geminivirus infection, with reduced symptoms in tolerant vs. susceptible cultivars [11,62]. This underscores the potential role of WRKY57 in modulating the defense response against SLCMV infection. The intricate interplay between WRKY57 and other proteins indicates its contribution to the differential gene expression between the tolerant and susceptible cultivars. Thus, *WRKY57* upregulation at 32 dpi in KU 50 may be linked to the defense mechanism against SLCMV infection and the resulting reduced CMD symptoms.

*WRKY75* (which was uniquely expressed in R 11, up- and downregulated at 32 and 67 dpi, respectively) regulates the ABA-dependent signaling pathway and is related to plant development, with downregulated WRKY75 decreasing ABA-dependent signaling [35,36]. In *Arabidopsis*, *WRKY75* downregulation decreases the effects of ABA, while upregulation increases ABA accumulation [35]. Additionally, WRKY75 interacts with and is repressed by sigma factor binding protein 1 (SIB1), thereby increasing PTI immunity (Figure 5) [35,94]. Therefore, the upregulation and then downregulation of *WRKY75* in R 11 may enhance susceptibility to SLCMV infection due to the complex regulation mediated by *WRKY75* expression.

*WRKY39* (which was prominently upregulated at 32 dpi in KU 50 and at 67 dpi in R 11) is involved in abiotic stress responses. WRKY39 interacts with ethylene overproducer 1 (ETO1), ADP-glucose (ADG1), isoamylase 1 (ISA1), MYB domain protein 36 (MYB36), and calmodulin 2 and 5 (CAM2 and 5) (Figure 5), and it is involved in heat tolerance in *A. thaliana* [57]. Furthermore, WRKY47 interacts with radical-induced cell death 1 (RCD1) (Figure 5), which is a crucial regulator in *A. thaliana*, influencing various aspects such as ROS production, programmed cell death prevention, hormonal and developmental responses, and abiotic stress responses, including improving salt tolerance [95,96,97]. The earlier expression of *WRKY47* (at 32 dpi; middle/recovery stage) in KU 50 may contribute to the defense response to SLCMV in KU 50, possibly indicating robustly restricted viral multiplication in this tolerant cultivar, which could ameliorate CMD.

**Figure 6:**
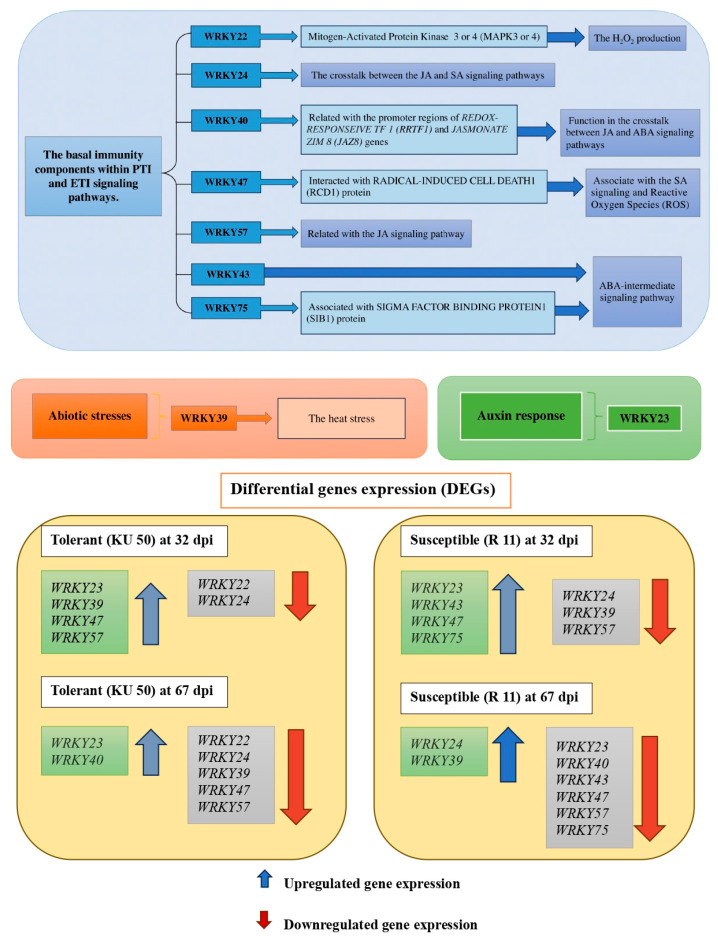
Summary of functions and interacting proteins of nine selected WRKY DEGs at 32 and 67 dpi in SLCMV-infected KU 50 (tolerant) and R 11 (susceptible). Blue, orange, and green boxes represent WRKYs involved in basal immunity (such as PTI and ETI pathways), abiotic stress responses, and other hormones, respectively. Regarding the blue boxes, the seven WRKYs (*WRKY22*, *-24*, *-40*, *-43*, *-47*, *-57*, and *-75*) participate in plant basal immunity responses, interacting with the ETI or PTI signaling pathways. Arrows represent the interacting proteins/corresponding genes and the plant responses. These WRKYs are up- and downregulated in response to SLCMV infection. Regarding the orange and green boxes, *WRKY23* and *-39* regulate abiotic stress responses and hormone biosynthesis, respectively.

Figure 6 displays an overview of the functions of nine selected WRKYs during SLCMV infection in KU 50 and R 11, combined with the expression of the WRKYs, their interacting partners, and the resultant plant responses. As the interacting partners are generally intermediates in complex pathways, the findings can be interpreted as the WRKYs indirectly influencing the plant responses via their effects on intermediates. In other words, there is a complicated network involving many connected proteins, which work together to ultimately increase or decrease gene expression and bring about plant responses.

### 3.4. RT-qPCR Validation

RNA-seq data on nine WRKYs (*WRKY22*, *-23*, *-24*, *-39*, *-40*, *-43*, *-47*, *-57*, and -*75*, which participate in numerous plant defense, hormone, and metabolite pathways) were subjected to validation using RT-qPCR. Although the *WRKY* expression was often similar between the RT-qPCR (which quantifies the expression of a few genes of interest using specific primers) and RNA-seq (which analyzes the whole transcriptome) results, there were some differences, which might be due to several factors. RNA-seq involves mRNA enrichment, which can introduce biases, while RT-qPCR can be more sensitive and accurate, but primer specificity can be a concern [98,99,100]. Additionally, cDNA, which is essential in the RT-qPCR technique, is derived from RNA templates [101], and degraded RNA can impact the quantitation of expression levels, potentially leading to bias. RNA-seq involves the entire spectrum of expressed genes, including both undegraded and degraded RNA.

## 4. Materials and Methods

### 4.1. Plant Materials, SLCMV Inoculation, and Leaf Sample Collection

Non-SLCMV-infected stems of Thai cassava (*M. esculenta*) cultivars KU 50 and R 11 (obtained from the Thai Tapioca Development Institute, Thailand) were selected to represent tolerant and susceptible phenotypes, respectively. They were planted in a greenhouse at the Department of Plant Pathology, Faculty of Agriculture, Kasetsart University, Thailand, and grown at 27–29 °C, 14 h light, and 70–80% humidity.

Six weeks after planting, SLCMV inoculation was performed by grafting SLCMV-infected rootstocks and healthy KU 50 and R 11 scions (three biological replicates), as previously described by Hemniam et al. (2019) [12]. Whether the scions and rootstocks were infected with SLCMV or healthy was confirmed using the PCR technique described below. The two or three leaves of each rootstock were maintained until new leaves emerged. CMD symptoms appeared at approximately 20 dpi. Leaves were collected at 21, 32, and 67 dpi and immediately stored in liquid nitrogen at −80 °C until use (Figure S1).

Next, cetyl trimethyl ammonium bromide (CTAB) DNA extraction [102] was conducted, and the plants were screened for SLCMV by polymerase chain reaction (PCR). The DNA quantity and quality were determined using a NanoDrop spectrophotometer (NanoDrop Technologies, Thermo Fisher Scientific, Waltham, MA, USA). The SLCMV *AV1* gene fragment was then PCR-amplified using specific forward (5′-GTT GAA GGT ACT TAT TCC C-3′) and reverse (5′-TAT TAA TAC GGT TGT AAA CGC-3′) primers [4]. The PCR products were visualized using 1.5% agarose TAE gel electrophoresis (100 V for 30 min) involving RedSafe Nucleic Acid Staining Solution (iNtRON Biotechnology, Sangdaewon, Republic of Korea) in 0.5x TAE buffer (1M Tris/HCl pH8, 0.5M ethylenediaminetetraacetic acid [EDTA], and glacial acetic acid). Finally, the gel images were analyzed using SYNGENE software (https://www.syngene.com/) (Synoptics Ltd., Cambridge, UK).

### 4.2. RNA Extraction and cDNA Library Construction

RNA was extracted from the leaves collected at 21, 32, and 67 dpi, as previously described [103]. The extracted RNA was resuspended in ultrapure nuclease-free water (20–100 μL, depending on the yield) and stored at −80 °C until use. RNA purity was evaluated based on absorbance ratios (A260:A280 and A260:A230) using a NanoDrop^®^ ND-1000 spectrophotometer (Thermo Fisher Scientific, Waltham, MA, USA). RNA quality was assessed by 1.5% agarose TAE gel electrophoresis (100 V for 30 min) involving RedSafe Nucleic Acid Staining Solution (iNtRON Biotechnology, Sangdaewon, Republic of Korea) in 0.5x TAE buffer (1M Tris/HCl pH8, 0.5M EDTA, and glacial acetic acid). A 1-kb DNA ladder (Thermo Scientific, USA) was also used. The gel images were then analyzed using SYNGENE software (Synoptics Ltd., Cambridge, UK).

A cDNA library was constructed using 1 µL RevertAid reverse transcriptase (Thermo Fisher Scientific, Waltham, MA, USA), along with 0.5 µL RiboLock RNase Inhibitor, 2 µL 10 mM dNTP mix, 1 µL 10 mM Oligo(dT), 4 µL 5x buffer, 10.5 µL water, and 1 µL 100 ng/µL template. The cDNA products were stored at −20 °C until use.

### 4.3. RNA-Seq and WRKY Identification

RNA-seq was conducted by Novogene Co., Ltd. (Beijing, China) using Illumina NovaSeq 6000 platforms. The raw FASTQ data on SLCMV-infected KU 50 and R 11 at 21, 32, and 67 dpi can be obtained from the Sequence Read Archive (SRA) under the BioProject accession number PRJNA1040252.

To identify WRKYs in SLCMV-infected KU 50 and R 11 at 21, 32, and 67 dpi, the data were used to conduct BLASTn searches (https://blast.ncbi.nlm.nih.gov/Blast.cgi, accessed on 19 November 2022) against the *M. esculenta*_v8 genome assembly from the NCBI database (RefSeq accession number GCF_001659605.2; https://www.ncbi.nlm.nih.gov/; accessed on 30 November 2023). The identified WRKYs, along with their expression levels (quantified by using Salmon v1.10.1) at the three time points, are provided in Table S1.

### 4.4. WRKY Functional Annotation

The complete amino acid sequences for each accession number were obtained from the NCBI database. The limited information on the WRKY family in cassava necessitates comparisons with homologs in well-annotated model plants, such as *A. thaliana* (AtWRKYs). To functionally annotate our identified WRKYs, complete amino acid sequences were obtained from The *Arabidopsis* Information Resource (TAIR; https://www.arabidopsis.org/browse/gene_family/WRKY, accessed on 21 November 2022). Multiple amino acid sequence alignments confirmed the conserved domains of our identified WRKY in relation to the *A. thaliana* homologs (AtWRKYs) (Table S2).

### 4.5. Phylogenetic Tree Construction

The NGPhylogeny platform [104] was used to construct a phylogenetic tree of our identified WRKYs and the *A. thaliana* WRKYs (AtWRKYs) based on the phylogenetic tree reported by Wang et al. (2023) [59]. Adjustment was conducted using the Interactive Tree of Life (ITOL) tool (bootstrap values were superimposed on branches with <50% support). The WRKYs clustered in the tree according to their homologous amino acid sequences, including their zinc finger motifs, as highlighted in a previously described classification [3,21,22].

### 4.6. WRKY Differentially Expressed Genes (DEGs)

DESeq2 in RStudio v4.1.2 was used to identify the significant *WRKY* DEGs (*p* < 0.01 and log_2_(fold change) ≤ 1.0) for 21 to 32 dpi (early to middle infection stages) 32 to 67 dpi (middle to late infection stages) in SLCMV-infected KU 50 and R 11 (Table S3). The up- and downregulated *WRKY* DEGs were visualized in Venn diagrams constructed using jvenn (http://jvenn.toulouse.inra.fr/app/index.html, accessed on 20 February 2023) [105]. The functions of the up- and downregulated *WRKY* DEGs at 32 and 67 dpi in KU 50 and R 11 were then explored. A heatmap of the *WRKY* DEGs was created using MetaboAnalyst based on Ward clustering involving the Euclidean distance metric.

### 4.7. RT-qPCR Validation

The RNA-seq results of nine significantly up- or downregulated WRKYs (including three that were uniquely expressed DEGs in a specific cultivar) were validated by RT-qPCR. Primers for these WRKYs were designed using Primer3 [106] and BLAST via NCBI (Table S4). A cDNA library was constructed (as described above) from the total RNAs from SLCMV-infected KU 50 and R 11 at 21, 32, and 67 dpi. qPCR amplification was conducted on a CFX96 Real-Time PCR detection system (Bio-Rad, Hercules, CA, USA) using 0.5 µL of each primer (forward and reverse primers were reconstituted with RNase-free water to a concentration of 1 pmol/µL), 3 µL nuclease-free water, 1 µL 100 ng/mL cDNA template, and lastly, 5 µL qPCRBIO 100x SyGreen Mix Lo-ROX (Copenhagen Biotech Supply, Bronshoj, Denmark). The relative gene expression (log2^−∆CQ^) was determined based on cycle quantities (Cq), i.e., ∆CQ (Cq of the control time point—Cq of the “experimental” time point) to compare 21 to 32 dpi and 32 to 67 dpi in SLCMV-infected susceptible and tolerant cultivars.

### 4.8. Interacting Partners of WRKYs

The interacting partners of the WRKYs were then determined using STITCH v5 [107] based on protein interactions derived from *A. thaliana* data.

## 5. Conclusions

The WRKY family in cassava is not well-annotated, so our results were elucidated by analyzing the functions of AtWRKYs homologs. This study involved determining the functional annotation and assessing the interacting partners of the WRKYs, aiming to enhance our understanding of SLCMV-infected KU 50 and R 11 across three time points (21, 32, and 67 dpi). Through phylogenetic analysis of our identified WRKYs and AtWRKYs, followed by functional annotation, we examined the conservation of amino acid sequences to shed light on the WRKYs’ roles in transcriptional regulation during various stages of SLCMV infection. This comprehensive investigation allowed us to discern the dynamic regulation of WRKYs, which enable cassava to adapt and respond flexibly to biotic and abiotic stressors. Certain WRKYs exhibited positive regulation during the middle/recovery stage (32 dpi) in SLCMV-infected KU 50, correlating with reduced CMD symptoms. Additionally, certain WRKYs in both cultivars displayed defense functions associated with mitigating disease severity. This highlights the pivotal role of WRKYs in regulating cassava defense mechanisms and underscores their significance in determining tolerance/susceptibility to SLCMV. Our research elucidated the regulatory mechanisms of WRKYs involving various cassava defense pathways, influencing the phenotypic differences between tolerant and susceptible cultivars. These mechanisms involve the modulation of SA and JA signaling pathways in addition to ROS production and functions related to other hormones and metabolites. This research contributes valuable insights to the topic of disease resistance in cassava and lays a foundation for future molecular breeding efforts, including the development of genetic markers of enhanced disease resistance.

## Figures and Tables

**Table 1:** List of identified WRKYs, along with their functional classification and their homolog ontology, based on alignment with the amino acid sequences of *Arabidopsis thaliana* WRKYs (AtWRKYs).

NCBI Accession Numbers	Gene ID	WRKYs Family	AtWRKYs * (*Arabidopsis thaliana* Homolog)	Homolog Functions
**KU 50**				
**Plant defenses**				
XM_021762774.1	110619370	WRKY DNA-binding protein 22	AtWRKY14, -22, -29	Coordinating with the MAPK signaling pathway and related to H_2_O_2_ enhancement [31,32,33]
**R 11**				
**Other related hormones and further abiotic stress response**				
XM_021756782.2	110615072	WRKY DNA-binding protein 43	AtWRKY24, -43, -56	Regulation of the ABA-dependent gene expression [34]
XM_021743059.2	110604766	WRKY DNA-binding protein 75	AtWRKY24, -43, -56, -75	Regulation of the ABA intermediate signaling pathway in *Arabidopsis* [35]
**Plant developments**				
XM_021743059.2	110604766	WRKY DNA-binding protein 75	AtWRKY24, -43, -56, -75	Related to phosphate accumulation within plant growth and development [36]
**KU 50 and R 11**				
**Hormone signaling (abscisic acid, SA, and JA)**				
XM_021774985.2	110628349	WRKY DNA-binding protein 1	AtWRKY1	Suppressing SA signaling [37]
XM_021778063.2	110630526	WRKY DNA-binding protein 4	AtWRKY3, -4, -44	Responding to JA stresses in *Arabidopsis* [38]
XM_021761541.2	110618414	WRKY DNA-binding protein 9	AtWRKY9, -72	Utilizing SA-independent defense mechanisms [27,39]
XM_021779074.2	110631303	WRKY DNA-binding protein 14	AtWRKY14, -22, -27, -29	Responding to SA and JA pathways in biotic and abiotic interruptions [40].
XM_021759593.2	110617016	WRKY DNA-binding protein 27	AtWRKY22, -27, -29, -14	Modulating the roles of the SA and JA pathways [40].
XM_021742749.2	110604542	WRKY DNA-binding protein 28	AtWRKY28, -57	Associated with JA signaling pathway [23]
XM_021742177.2	110604092	WRKY DNA-binding protein 31	AtWRKY31, -36, -42, -47	Regulation through the modulation of SA signaling [33]
XM_021765491.2	110621275	WRKY DNA-binding protein 44	AtWRKY3, -4, -44	Activating the tolerances within JA stresses in *Arabidopsis* [38]
XM_021756034.2	110614486	WRKY DNA-binding protein 47	AtWRKY31, -36, -42, -47	Regulation through the modulation of SA signaling [33]
XM_021738919.2	110601688	WRKY DNA-binding protein 48	AtWRKY23, -28, -57, -48	Associated with SA regulation by induce the PR1 in the bacterial pathogen infection [41]
XM_021774174.2	110627806	WRKY DNA-binding protein 51	AtWRKY24, -43, -51, -56, -75	Intermediating the SA; otherwise, repressing JA signaling [42,43]
XM_021748704.2	110609254	WRKY DNA-binding protein 53	AtWRKY41, -53, -55	Related to SA signaling induction of *Arabidopsis* [23]
XM_021761582.2	110618450	WRKY DNA-binding protein 55	AtWRKY41, -53, -55	Regulating the SA signaling pathway [44]
XM_043957127.1	110614243	WRKY DNA-binding protein 57	AtWRKY28, -57	Regulating the JA signaling pathway in case of fungal infection [45]
XM_021740051.2	110602513	WRKY DNA-binding protein 72	AtWRKY9, -72	Utilizing SA-independent defense mechanisms [39]
**Reactive Oxygen Species (ROS)**				
XM_021742177.2	110604092	WRKY DNA-binding protein 31	AtWRKY31, -36, -42, -47	Regulating of ROS synthesis [33]
XM_021756034.2	110614486	WRKY DNA-binding protein 47	AtWRKY31, -36, -42, -47	Regulation through ROS synthesis [33]
XM_021761582.2	110618450	WRKY DNA-binding protein 55	AtWRKY41, -53, -55	Regulation of ROS accumulation [44]
**Basal immune mechanisms**				
XM_021774985.2	110628349	WRKY DNA-binding protein 1	AtWRKY1	Related to the pathogenesis-related (PR) proteins stimulated [37]
XM_021758490.1	110616151	WRKY DNA-binding protein 7	AtWRKY7, -15	Regulating plant defense against bacterial pathogens and triggering the HR, which eventually induces cell death programming [27,29]
XM_021761541.2	110618414	WRKY DNA-binding protein 9	AtWRKY9, -72	Contributing to the plant basal defense against bacteria and nematode pathogens and coordinating the elicited HR mechanism [39]
XM_021756448.2	110614786	WRKY DNA-binding protein 12	AtWRKY12, -13	Regulated positively in the plant defense mechanism [23]
XM_021751457.2	110611253	WRKY DNA-binding protein 21	AtWRKY15, -17, -21, -39	Controlling plant defense signaling against bacterial infection [46,47]
XM_021757556.2	110615593	WRKY DNA-binding protein 23	AtWRKY23, -48	Accompanying an avirulent-to-bacterial infection [48,49]
XM_021751071.2	110610985	WRKY DNA-binding protein 24	AtWRKY24, -43, -51, -56, -75	Has a role in the basal immunity (PTI) expression of the early defense response in *Oryza sativa* ssp. *indica* [50]
XM_021749661.2	110609837	WRKY DNA-binding protein 26	AtWRKY2, -26, -33	Regulating resistance to necrotrophic pathogens [51,52]
XM_021759593.2	110617016	WRKY DNA-binding protein 27	AtWRKY22, -27, -29, -14	Involved in pathogen-triggered immunity [40]
XM_021767775.2	110622985	WRKY DNA-binding protein 33	AtWRKY2, -26, -33	Regulating plant-induced resistance to the necrotrophic pathogens [51,52]
XM_021738919.2	110601688	WRKY DNA-binding protein 48	AtWRKY23, -28, -57, -48	Influencing the plant basal resistance associated with PR1 in the bacterial pathogen infection [41]
XM_021766075.2	110621777	WRKY DNA-binding protein 49	AtWRKY49	Related to resistance and increasing defense gene expression [53]
XM_021761805.2	110618630	WRKY DNA-binding protein 70	AtWRKY70	Corresponds to the NPR1 protein and is related to enhancing PR1 gene expression [54,55]
XM_021740051.2	110602513	WRKY DNA-binding protein 72	AtWRKY9, -72	Utilizing plant basal immunity [39]
**Other related hormones, metabolites, and abiotic stress responses**				
XM_021757679.2	110615677	WRKY DNA-binding protein 2	AtWRKY2, -26, -33	Enhanced during heat stress [51,52]
XM_021776299.2	110629365	WRKY DNA-binding protein 15	AtWRKY15, -17, -21, -39	Enhancing plant metabolite pathways [56]
XM_021741844.2	110603865	WRKY DNA-binding protein 17	AtWRKY17, -15, -21, -39	Responding to drought stress in bacterial infection [46,47]
XM_021757556.2	110615593	WRKY DNA-binding protein 23	AtWRKY23, -48	Responding to auxin hormones in nematode resistance [48,49]
XM_021749661.2	110609837	WRKY DNA-binding protein 26	AtWRKY2, -26, -33	Enhanced during heat stress [51,52]
XM_021742749.2	110604542	WRKY DNA-binding protein 28	AtWRKY28, -57	Associated with ABA hormone transcriptional regulation [23]
XM_021767775.2	110622985	WRKY DNA-binding protein 33	AtWRKY2, -26, -33	Enhanced during heat stress [51,52]
XM_021766598.2	110622177	WRKY DNA-binding protein 39	AtWRKY15, -17, -21, -39	Functions in the ethylene hormone and heat tolerant in *Arabidopsis thaliana* [57]
XM_021772630.2	110626614	WRKY DNA-binding protein 40	AtWRKY18, -40, -60	Regulating the intermediating of the ABA hormone signaling pathway [23]
XM_043950216.1	110631349	WRKY DNA-binding protein 41	AtWRKY41, -53, -55	Regulating plant general hormone signaling and response to biotic stresses [23]
XM_021759626.2	110617045	WRKY DNA-binding protein 56	AtWRKY24, -43, -51, -56, -75	Responding to salt stress in *Arabidopsis thaliana* [58]

* The AtWRKYs homolog annotated from the *Arabidopsis* Information Resource (TAIR) by using amino acid sequence alignments confirmed the conserved domains of our identified WRKY in relation.

**Table 2:** WRKY differentially expressed genes (DEGs; *p* < 0.01 and log_2_(fold change) ≤ 1.0) at 32 and 67 dpi in SLCMV-infected R 11 and KU 50.

NCBI Accession Numbers	Gene ID	WRKYs Families	KU 50				R 11			
			32 dpi		67 dpi		32 dpi		67 dpi	
			DEGs	*p*-Value	DEGs	*p*-Value	DEGs	*p*-Value	DEGs	*p*-Value
XM_021774985.2	110628349	WRKY DNA-binding protein 1	−0.88	0.58	ND *	ND	−2.30	0.53	ND	ND
XM_021757679.2	110615677	WRKY DNA-binding protein 2	ND	ND	−1.10	0.61	0.32	0.76	−3.80	0.02
XM_021778063.2	110630526	WRKY DNA-binding protein 4	−0.03	0.96	−0.69	0.40	0.29	0.64	−1.04	0.08
XM_021758490.1	110616151	WRKY DNA-binding protein 7	−0.94	0.04	−0.01	0.99	−1.01	0.07	−0.34	0.54
XM_021761541.2	110618414	WRKY DNA-binding protein 9	ND	ND	ND	ND	ND	ND	ND	ND
XM_021756448.2	110614786	WRKY DNA-binding protein 12	ND	ND	ND	ND	ND	ND	ND	ND
XM_021779074.2	110631303	WRKY DNA-binding protein 14	ND	ND	ND	ND	ND	ND	ND	ND
XM_021776299.2	110629365	WRKY DNA-binding protein 15	−1.03	0.1	0.51	0.58	−1.71	0.01	1.04	0.09
XM_021741844.2	110603865	WRKY DNA-binding protein 17	ND	ND	0.14	0.69	ND	ND	−0.4	0.17
XM_021751457.2	110611253	WRKY DNA-binding protein 21	0.32	0.5	0.03	0.96	0.53	0.37	−1.40	0.01
XM_021762774.1	110619370	WRKY DNA-binding protein 22	−1.69	0.02	−0.22	0.87	ND	ND	ND	ND
XM_021757556.2	110615593	WRKY DNA-binding protein 23	2.83	0.32	1.12	0.53	1.10	0.79	−0.78	0.85
XM_021751071.2	110610985	WRKY DNA-binding protein 24	0.93	0.82	0.32	0.94	−2.30	0.56	1.14	0.78
XM_021749661.2	110609837	WRKY DNA-binding protein 26	ND	ND	−0.27	0.88	ND	ND	−3.26	0.24
XM_021759593.2	110617016	WRKY DNA-binding protein 27	ND	ND	ND	ND	ND	ND	ND	ND
XM_021742749.2	110604542	WRKY DNA-binding protein 28	ND	ND	ND	ND	ND	ND	ND	ND
XM_021742177.2	110604092	WRKY DNA-binding protein 31	ND	ND	−2.39	0.19	−1.67	0.08	−0.56	0.67
XM_021767775.2	110622985	WRKY DNA-binding protein 33	ND	ND	−2.13	0.58	ND	ND	−2.26	0.53
XM_021766598.2	110622177	WRKY DNA-binding protein 39	2.19	0.09	−0.03	0.98	−0.18	0.92	1.18	0.47
XM_021772630.2	110626614	WRKY DNA-binding protein 40	ND	ND	2.45	0.05	ND	ND	−0.23	0.87
XM_043950216.1	110631349	WRKY DNA-binding protein 41	ND	ND	ND	ND	ND	ND	ND	ND
XM_021756782.2	110615072	WRKY DNA-binding protein 43	ND	ND	ND	ND	1.10	0.79	−0.78	0.85
XM_021765491.2	110621275	WRKY DNA-binding protein 44	ND	ND	0.75	0.04	0.21	0.48	ND	ND
XM_021756034.2	110614486	WRKY DNA-binding protein 47	1.47	0.16	−0.07	0.95	3.80	0.14	0.83	0.48
XM_021738919.2	110601688	WRKY DNA-binding protein 48	0.97	0.63	0.32	0.88	−1.34	0.65	−0.78	0.85
XM_021766075.2	110621777	WRKY DNA-binding protein 49	ND	ND	ND	ND	ND	ND	ND	ND
XM_021774174.2	110627806	WRKY DNA-binding protein 51	0.61	0.32	−1.39	0.13	−1.57	0.03	−0.26	0.75
XM_021748704.2	110609254	WRKY DNA-binding protein 53	ND	ND	−0.69	0.73	ND	ND	−3.26	0.25
XM_021761582.2	110618450	WRKY DNA-binding protein 55	ND	ND	ND	ND	ND	ND	ND	ND
XM_021759626.2	110617045	WRKY DNA-binding protein 56	ND	ND	ND	ND	ND	ND	ND	ND
XM_043957127.1	110614243	WRKY DNA-binding protein 57	3.15	0.23	−1.01	0.67	−1.76	0.52	−0.78	0.85
XM_021761805.2	110618630	WRKY DNA-binding protein 70	0.12	0.85	−1.06	0.28	ND	ND	−2.46	0.02
XM_021740051.2	110602513	WRKY DNA-binding protein 72	ND	ND	ND	ND	ND	ND	ND	ND
XM_021743059.2	110604766	WRKY DNA-binding protein 75	ND	ND	ND	ND	1.04	0.78	−1.68	0.68

* ND = not determined.

**Table 3:** Comparison of WRKY expression between RNA-seq and RT-qPCR data (log2^−∆CQ^) in KU 50 and R 11 at 32 and 67 dpi.

NCBI Accession Numbers	Name of WRKYs Transcription Factors	R 11				KU 50			
		32 dpi		67 dpi		32 dpi		67 dpi	
		DEGs	log 2^∆CQ^	DEGs	log 2^∆CQ^	DEGs	log 2^∆CQ^	DEGs	log 2^∆CQ^
XM_021762774.1	WRKY22	ND *	−0.11	ND	0.01	−1.69	−0.54	−0.22	0.13
XM_021757556.2	WRKY23	1.10	0.51	−0.78	0.67	2.83	−0.24	1.12	−0.12
XM_021751071.2	WRKY24	−2.30	1.34	1.14	0.61	0.93	−0.35	0.31	−0.01
XM_021766598.2	WRKY39	−0.18	0.94	1.18	0.59	2.19	−0.31	−0.03	−0.13
XM_021772630.2	WRKY40	ND	0.60	−0.23	0.71	ND	−1.24	2.45	−0.30
XM_021756782.2	WRKY43	1.10	0.02	−0.78	1.26	ND	−0.42	ND	0.17
XM_021756034.2	WRKY47	3.80	−0.01	0.83	0.01	1.47	0.09	−0.07	−0.03
XM_043957127.1	WRKY57	−1.76	1.20	−0.78	0.91	3.15	−0.32	−1.01	−0.04
XM_021743059.2	WRKY75	1.04	0.71	−1.68	0.50	ND	−0.19	ND	−0.25

* ND = not determined.

## Data Availability

The datasets generated and analyzed in the current study are available in the NCBI database under the Sequence Read Archive (SRA) accession number PRJNA1040252, and the datasets supporting the conclusions of this article are included with the article and its Supplementary Materials.

## Supplementary Materials

The following supporting information can be downloaded at https://www.mdpi.com/article/10.3390/plants14081159/s1, Figure S1: Plant sampling of KU 50 (tolerant) and R 11 (susceptible) cultivars following graft inoculation. CMD symptoms were distinct between the two cultivars: KU 50 exhibited mild symptoms on newly emerged leaves, while R 11 showed severe symptoms, including mosaic patterns and leaf distortion; Table S1: Raw data on the expression of 34 identified WRKYs in SLCMV-infected KU 50 and R 11 at 21, 32, and 67 dpi; Table S2: Raw data on protein sequences of 34 identified WRKYs and AtWRKYs (Arabidopsis thaliana WRKYs), obtained from the NCBI and TAIR database, respectively; Table S3: Raw data on the expression of WRKY differentially expressed genes (DEGs; *p* < 0.01 and log2(fold change) ≤ 1.0) in SLCMV-infected KU 50 and R 11 at 32 and 67 dpi; Table S4: Primer sequences for RT-qPCR validation of nine selected WRKYs.
